# Particulate Matter Induces Oxidative Stress and Ferroptosis in Human Lung Epithelial Cells

**DOI:** 10.3390/toxics12020161

**Published:** 2024-02-19

**Authors:** Yujin Ahn, Yong-Hyeon Yim, Hee Min Yoo

**Affiliations:** 1 Biometrology Group, Korea Research Institute of Standards and Science (KRISS), Daejeon 34113, Republic of Korea; 2 Department of Precision Measurement, University of Science and Technology (UST), Daejeon 34113, Republic of Korea; 3 Inorganic Metrology Group, Korea Research Institute of Standards and Science (KRISS), Daejeon 34113, Republic of Korea

**Keywords:** particulate matter, air pollution, cytotoxicity, ROS, mitochondrial dysfunction, cell death, ferroptosis

## Abstract

Numerous toxicological studies have highlighted the association between urban particulate matter (PM) and increased respiratory infections and lung diseases. The adverse impact on the lungs is directly linked to the complex composition of particulate matter, initiating reactive oxygen species (ROS) production and consequent lipid peroxidation. Excessive ROS, particularly within mitochondria, can destroy subcellular organelles through various pathways. In this study, we confirmed the induction of ferroptosis, an iron-dependent cell death, upon exposure to an urban PM using RT-qPCR and signaling pathway analysis. We used KRISS CRM 109-02-004, the certified reference material for the analysis of particulate matter, produced by the Korea Research Institute of Standards and Science (KRISS). To validate that ferroptosis causes lung endothelial toxicity, we assessed intracellular mitochondrial potential, ROS overproduction, lipid peroxidation, and specific ferroptosis biomarkers. Following exposure to the urban PM, a significant increase in ROS generation and a decrease in mitochondrial potential were observed. Furthermore, it induced hallmarks of ferroptosis, including the accumulation of lipid peroxidation, the loss of antioxidant defenses, and cellular iron accumulation. In addition, the occurrence of oxidative stress as a key feature of ferroptosis was confirmed by increased expression levels of specific oxidative stress markers such as NQO1, CYP1B1, FTH1, SOD2, and NRF. Finally, a significant increase in key ferroptosis markers was observed, including xCT/SLC7A11, NQO1, TRIM16, HMOX-1, FTL, FTH1, CYP1B1, CHAC1, and GPX4. This provides evidence that elevated ROS levels induce oxidative stress, which ultimately triggers ferroptosis. In conclusion, our results show that the urban PM, KRISS CRM, induces cellular and mitochondrial ROS production, leading to oxidative stress and subsequent ferroptosis. These results suggest that it may induce ferroptosis through ROS generation and may offer potential strategies for the treatment of lung diseases.

## 1. Introduction

Exposure to particulate matter (PM) is strongly associated with adverse health effects, particularly in the lungs [1]. It can cause short-term effects such as irritation of the eyes, nose, throat, and lungs, and symptoms such as coughing, sneezing, and shortness of breath [2]. In addition, PM, especially fine particulate matter of 2.5 μm or smaller (PM2.5), can penetrate deep into the respiratory tract and reach the lungs, contributing to reduced lung function and disease [3,4]. Airborne particles are categorized based on their size, chemical composition, and shape. Those with a diameter of 2.5 μm or less are termed fine particulate matter (PM2.5), constituting a fraction of PM10 [5]. This study used certified reference material (CRM) of PM provided by the Korea Research Institute of Standards and Science (KRISS). The material obtained through the sieve passing PM2.5 and PM10 serves as a critical component of our analysis (Supplementary Tables S1 and S2).

Given the numerous chemical components in ambient aerosols, the specific damage associated with each particle type based on elemental composition remains uncertain [6]. However, it is widely recognized that PM can induce toxic effects such as DNA damage, DNA methylation, and genotoxicity [7]. To investigate the relationship between ambient PM and adverse effects on lung health, we used KRISS CRM 109-02-004, derived from the urban environment. It can be a valuable tool for assessing performance and validation. Several studies have proposed a significant correlation between PM and oxidative stress in the lungs, yet the precise mechanisms remain elusive [8,9]. In this study, we confirmed that exposure to PM can induce oxidative stress through excessive reactive oxygen species (ROS) and lipid peroxidation, which plays a key role in a novel form of cell death, ferroptosis.

Ferroptosis, introduced by Dixon in 2012, is an iron-dependent form of programmed cell death triggered by a redox imbalance between oxidants and antioxidants [10,11,12]. Unlike other cell death mechanisms, ferroptosis involves both ROS and iron, leading to distinct morphological changes in mitochondria, increased membrane density, and the release of lipid metabolites from the cell body [13,14,15]. Ferroptosis, which is involved in various cellular functions, presents a promising strategy for treating lung diseases through targeted modulation of oxidative-stress-related pathways [16]. Oxidative stress, causing neuronal dysfunction and cell death, results from an imbalance between ROS production and antioxidant depletion [17]. This imbalance further contributes to ferroptosis, characterized by inefficient lipid peroxide clearance and the presence of excessive iron [18,19]. Iron-dependent lipid peroxidation, regulated by glutathione peroxidase 4 (GPX4), is a crucial factor in driving ferroptosis [20]. GPX4 detects and responds to oxidative stress, with glutathione depletion as one of its markers, associated with DNA damage, excess ROS, and cellular toxicity [21,22]. Furthermore, reduced activity of antioxidant enzymes, including superoxide dismutase (SOD), heme oxygenase-1 (HMOX-1), catalase (CAT), and glutathione peroxidase (GSH-Px), plays a key role in oxidative-stress-induced lung damage [23]. In the complex cellular response network, NRF2 plays a central role in coordinating the antioxidant defense system against oxidative stress [24,25]. Many studies have highlighted NRF2’s regulatory impact on GPX4, underlining its importance in cellular defense mechanisms [26,27]. Further in-depth investigation into the molecular mechanisms of this pathway is essential, as increased oxidative stress may be an indicator of several diseases such as diabetes, cancer, and neurodegenerative disorders [28,29].

In the realm of ferroptosis, the crucial player xCT/SLC7A11 shows a significant correlation with GPX4 [30]. Reduced levels of xCT and GPX4 result in lower intracellular cysteine concentration, affecting glutathione synthesis and lipid peroxide degradation. In addition, NRF2 promotes ferroptosis by suppressing pro-ferroptotic genes involved in GSH production, ROS detoxification, and iron sequestration, such as FTH1, FTL, xCT/SLC7A11, NQO1, ACSL4, NOX4, HMOX-1, and GCLM, regulated through the NRF2/KEAP-1 pathway [31]. Notably, genes related to iron metabolism (FTH1, FTL, xCT/SLC7A11, HO-1) are commonly upregulated during ferroptosis via iron-responsive element-binding protein 2 (IREB2) [32]. HO-1 may play a dual role, as excessive heme promotes ferroptosis in cancer cells [33,34]. Additionally, the ferritin complex components FTL and FTH1 are upregulated with increased iron uptake [35,36]. Our study confirms the upregulation of pro-ferroptotic genes associated with iron metabolism, validating that particulate matter induces ferroptosis by promoting iron accumulation.

Numerous studies have distinguished ferroptosis from apoptosis, necrosis, and autophagy, often noting necrosis-like morphological changes [37,38]. Our research into the central role of mitochondria in ROS production, lipid peroxidation, iron metabolism, and ferroptosis led us to investigate the effects of particulate matter on mitochondrial function. Crucially, differentiating between cytosolic and mitochondrial ROS is vital for understanding their involvement in ferroptosis [39,40]. In the context of human and animal respiratory systems, lungs facilitate gas exchange, mediating oxygen transfer and releasing carbon dioxide [41,42]. The lung defense system, which is responsible for removing inhaled debris, can be compromised by air pollution, particularly airborne particulate matter (PM), which has been linked to increased lung cancer and mortality [43,44]. Understanding the mechanisms of damage leading to diseases such as lung cancer or pneumonia from PM exposure is essential.

The aim of this study was to confirm the cytotoxicity of KRISS CRM, a CRM for urban PM, and to investigate its effects on lung physiology and the mechanisms involved. Our results confirm the upregulation of pro-ferroptotic genes related to iron metabolism and provide evidence that PM induces ferroptosis through increased iron accumulation. We also observed elevated levels of lipid ROS and mitochondrial ROS, indicative of lipid peroxidation. Given the limited knowledge of the pulmonary effects of PM and the mechanisms leading to lung disease, and the lack of studies on the effects of different chemical compositions, we used KRISS CRM to describe the precise cytotoxic mechanisms following PM exposure. This research provides valuable insights into the molecular pathways involved in PM-induced lung damage. The identified upregulation of pro-ferroptotic genes and lipid peroxidation provides potential targets for therapeutic intervention to reduce the adverse effects of PM on lung health.

## 2. Materials and Methods

### 2.1. KRISS CRM Extract Preparation and Treatment

CRM for the analysis of urban PM was obtained from the Korea Research Institute of Standards and Science (KRISS, Daejeon, Republic of Korea). KRISS CRM 109-02-004 is an urban fine dust CRM certified for elements and mass fractions of polycyclic aromatic hydrocarbons (PAHs). The raw material for this CRM was collected from the intake air filtration systems of large buildings and facilities in the Seoul metropolitan area for two years (2019–2020). The particulate matter collected on the filters was recovered and combined into a single lot of about 6.2 kg. After the removal of coarse particles by multistage sieving, about 2.5 kg of fine dust particles passing through 20 µM sieves was obtained. The moisture content of the final product was less than 3%. Dimethyl sulfoxide (DMSO; Sigma-Aldrich, Merck, Darmstadt, Germany) was used to make a 10 mg/mL stock solution of PM and stored at −4 °C in the dark. The PM was treated in a culture medium.

### 2.2. Cell Culture and Treatment

The bronchial epithelial cell line, BEAS-2B cells, was obtained from the American Type Culture Collection (ATCC) (Manassas, VA, USA). The cells were maintained in Dulbecco’s modified Eagle’s medium (DMEM; Welgene, Republic of Korea) containing 10% fetal bovine serum (FBS; Welgene, Republic of Korea), and 1% penicillin–streptomycin (Thermo Fisher Scientific, Waltham, MA, USA) was used by the information suggested by KCLB or ATCC. The cells were cultured in a 5% CO_2_ incubator at 37 °C. Subcultures were performed with trypsin-EDTA solution when cell density reached 80–90%. Prior to exposure, the PM was pretreated in the medium to prevent aggregation, followed by thorough vortexing. Then, we added this mixture to the cells we had seeded the day before, similar to changing the media for cell cultures. Cells were harvested using trypsin-ethylenediaminetetraacetic acid (EDTA).

### 2.3. Cytotoxicity Assay

After 24 h, BEAS-2B cells were seeded in 96-well plates at a density of 1 × 10^4^ cells per well and subsequently exposed to KRISS CRM at concentrations of 50, 100, and 200 μg/mL. A cytotoxicity assay was assessed after 48 h of KRISS CRM treatment using the Cell Titer 96 AQueous One Solution Cell Proliferation Assay Kit (Promega, Madison, WI, USA). Using a Synergy HTX multimode microplate reader (BioTek Instruments, Inc., Winooski, VT, USA), the absorbance at 490 nm was measured after the cells had been treated with solution reagents for 3 h at 37 °C.

### 2.4. Cellular Morphology Microscopy

BEAS-2B cells were seeded in 6-well plates up to a density of 2 × 10^5^ cells in each well and subsequently treated with KRISS CRM. The KRISS CRM was treated after 24 h, and then after 48 h, cell morphology was observed under a phase 400× magnification contrast microscope (Olympus, Tokyo, Japan).

### 2.5. Reactive Oxygen Species’ (ROS’) Measurement 

BEAS-2B cells were treated with KRISS CRM for 48 h after being seeded in 6-well plates at a density of 2 × 10^5^ cells per well to measure reactive oxygen species. Intracellular ROS levels were measured using 2’, 7’-dichlorodihydrofluorescein diacetate acetyl ester (H_2_DCFDA) (Thermo Fisher Scientific, Waltham, MA, USA). The cells were incubated with H_2_DCFDA (1 µM) at room temperature for 40 min, then washed with phosphate buffer saline (PBS). Cells were suspended in FACS buffer (PBS supplemented with 1% PBS). Then, the intracellular ROS were examined using a flow cytometer (BD FACSVerse, BD Biosciences, San Jose, CA, USA) at an excitation wavelength of 500 nm. Each sample underwent the acquisition of 10,000 events, and the raw geometric mean fluorescence data were analyzed quantitatively using Flowjo software (Version 10, TreeStar, Ashland, OR, USA). Relative fluorescence was determined by normalizing to the control group.

### 2.6. Mitochondrial Membrane Potential (MMP, ∆ψm) Assay

To evaluate the mitochondrial membrane potential, the BEAS-2B cells were plated in 6-well plates at a density of 2 × 10^5^ cells per well. Then, the KRISS CRM was treated after 24 h, and cells were incubated for 48 h. The cells were then harvested and washed twice with cold PBS (Corning, Manassas, VA, USA). After that, cells were incubated with 100 nM TMRM (Thermo Fisher Scientific, Waltham, MA, USA) at 37 °C for 30 min. After incubation, the cells were once again washed, suspended in 2% FBS in PBS, and quantified using a flow cytometer (BD FACSVerse, BD Biosciences, San Jose, CA, USA) at an excitation wavelength of 488 nm. Each sample was processed with the acquisition of 10,000 events, followed by quantitative analysis of the raw geometric mean fluorescence data. Relative fluorescence was determined by normalizing to the control group.

### 2.7. Mitochondrial Superoxide Assay

To assess the mitochondrial superoxide levels of the cells, the BEAS-2B cells were seeded in 6-well plates up to a density of 2 × 10^5^ cells per well, and the cells were treated with KRISS CRM after 24 h. Cells were harvested after 48 h of incubation, washed two times with cold BioLegend Cell Staining Buffer, then resuspended in MitoSOX working solution buffer. We used the MitoSOX™ Mitochondrial Superoxide Indicators (Thermo Fisher Scientific, Waltham, MA, USA). The cells were treated with 10 µM MitoSOX solution, and they were incubated at room temperature in the dark for 30 min. For each sample, 10,000 events were collected and analyzed at an excitation wavelength of 510 nm. The experiments were performed independently a minimum of three times.

### 2.8. Lipid Peroxidation Assay

To index lipid peroxidation antioxidant efficacy using flow cytometry, BEAS-2B cells were plated in 6-well plates at a density of 2 × 10^5^ cells per well. KRISS CRM was then applied after 24 h and cells were incubated for 48 h. Lipid oxidation levels were measured using a lipophilic fluorescent dye 4,4-difluoro-5-(4-phenyl-1,3-butadienyl)-4-bora-3a,4a-diaza-s-indacene-3-undecanoic acid (C11-BODIPY^581/591^) to detect lipid oxidation induced by oxygen radicals by flow cytometry. The cells were incubated with C11-BODIPY^581/591^ for 30 min at room temperature and washed with PBS. Cells were suspended in FACS buffer (PBS supplemented with 1% PBS). Using a wavelength of 488 nm for BODIPY measurement, 10,000 cell events were measured for each sample, and analysis was performed using Flowjo software (version 10, Williamson, OR, USA). The gate was defined to encompass BODIPY-positive cells, with thresholds determined using fluorescence minus one (FMO) controls to differentiate specific staining from background fluorescence. The experiments were performed independently a minimum of three times.

### 2.9. Iron Accumulation Assay

Total iron levels were measured in wild type human lung epithelial cells (BEAS-2B cells) using an iron assay kit (Abcam, Cambridge, MA, USA) according to the manufacturer’s instructions. After lysing the samples in iron assay buffer, 5 μL of iron reducer were added to 50 μL of samples. Subsequently, 100 μL of iron probe solution was added to the samples and incubated at 37 °C for 30 min. After treatment with the iron probe, samples were mixed and further incubated for 60 min at 37 °C. Absorbance at a wavelength of 593 nm was then measured using spectrophotometry.

### 2.10. Quantitative Reverse Transcription PCR (RT-qPCR)

RNeasy mini kit (Qiagen, Hilden, Germany) was used to extract total RNA from BEAS-2B cells that were treated with KRISS CRM for 48 h. The RNA concentration was measured by Nanodrop (Thermo Fisher Scientific, Waltham, MA, USA). Then we synthesized cDNA using the iScript™ cDNA Synthesis Kit (Bio-Rad, Hercules, CA, USA) with an equivalent amount of RNA. A particular set of primers were added to the PCR reaction, comprising 10 µL of iTaq Universal SYBR Green Supermix (Bio-Rad, Hercules, CA, USA), 1 µL of PCR Forward Primer (10 µM), 1 µL of PCR Reverse Primer (10 µM), 1 µL of cDNA template, and 7 µL of ddH_2_O. A StepOnePlus Real-Time PCR system (Thermo Fisher Scientific, Waltham, MA, USA) was used for PCR reaction amplification. The reaction was amplified through a flowing step at 95 °C for 10 min of denaturation, followed by 40 cycles of 95 °C for 15 s, and 60 °C for 60 s. A primer sequence (5′→3′) was used in this experiment. The expression level of each gene was then calculated via the 2^−ΔΔCt^ method. The primer sequences for the genes used in RT-qPCR are provided (Supplementary Table S3).

### 2.11. Western Blot

To verify specific protein levels, BEAS-2B cells were seeded in a 6-well plate at a density of 2 × 10^5^ cells per well and treated with KRISS CRM for 48 h. Cells were washed twice with cold PBS and then lysed with RIPA buffer containing 0.1 mg/mL phenylmethylsulfonyl fluoride (PMSF), NEM, and protease inhibitor cocktail. The cell lysates were then centrifuged at 13,000 rpm at 4 °C. Protein quantification was performed using the Bradford assay on a Synergy HTX multimode microplate reader (BioTek Instruments, Inc., Winooski, VT, USA). Equal amounts of protein were separated by 10% SDS-PAGE before being transferred to a polyvinylidene difluoride (PVDF) membrane (Millipore Corp., Boston, MA, USA). Proteins were then probed with specific primary antibodies in Triton containing 3% BSA overnight at 4 °C in the dark. Specific primary antibodies against SOD1, SOD2, ACSL4, xCT, GPx-4, FTL, and the loading control proteins β-actin and GAPDH were purchased from Cell Signaling Technologies (Danvers, MA, USA) or Santa Cruz Biotechnology (Dallas, TX, USA). After primary antibody treatment, the membranes were washed with 1 × TBST and incubated with anti-mouse or anti-rabbit horseradish peroxidase-conjugated secondary antibodies for 2–3 h. Detection of protein signaling was performed using an Image Quant LAS mini (Fujifilm, Tokyo, Japan).

### 2.12. Statistical Analysis

GraphPad Prism (GraphPad Software, Inc., version 7, San Diego, CA, USA) was used for statistical analysis, and values were provided as means ± SEM. The Student’s *t*-test was used to evaluate the data. The obtained *p*-values (* *p* < 0.05, ** *p* < 0.01, *** *p* < 0.001, **** *p* ≤ 0.0001) were interpreted as statistically significant. 

## 3. Results

### 3.1. Cytotoxicity of the Certified Reference Material of Particulate Matter

**Figure 1:**
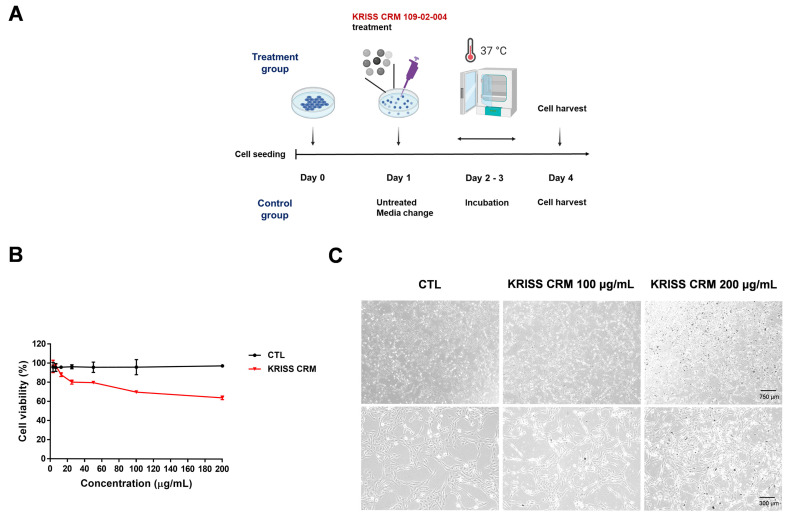
KRISS CRM induced cytotoxicity in BEAS-2B cells. (**A**) The schematic diagram of PM treatment experiment. Cells were incubated with 100 to 200 μg/mL KRISS CRM after 24 h of cell seeding. KRISS CRM for the indicated days, while control groups were untreated for the following 3 days. (**B**) Cell proliferation assay was measured using CellTiter 96^®^ Aqueous (Promega, Madison, WI, USA) on day 3 after PM treatment. (**C**) Cell morphology after 48 h of KRISS CRM treatment (scale bar: 750 μm and 300 μm).

To examine the cytotoxicity of the KRISS CRM of PM manufactured by KRISS, we used normal lung epithelial cells such as BEAS-2B. As shown in Figure 1A, PM was treated in these cells in 96-well plates for 48 h at concentrations between 0 and 200 μg/mL. As the concentration increased, cell viability decreased, reaching close to the IC60 at the concentration of 200 μg/mL as shown in Figure 1B. To examine the biological alterations in morphological responses upon particulate matter exposure in human lung epithelial cells, we conducted microscopic examination. Alterations in cellular morphology often indicate cellular stress or adaptation mechanisms, involving significant modifications in pathways associated with cell death, apoptosis, interactions with the extracellular matrix (ECM), and the structure of the cytoskeleton [45,46]. BEAS-2B cells were treated with 100 and 200 μg/mL of KRISS CRM for the microscopic analysis, resulting in induced cytotoxicity in a concentration-dependent manner (Figure 1C). In this microscopic analysis, we typically observed alterations such as changes in cell size, variations in cell morphology (such as elongation, rounding, or irregular shapes), the formation of cellular protrusions or extensions, and cell body contraction.

### 3.2. KRISS CRM Induces Cellular ROS Generation in Human Lung Cells

**Figure 2:**
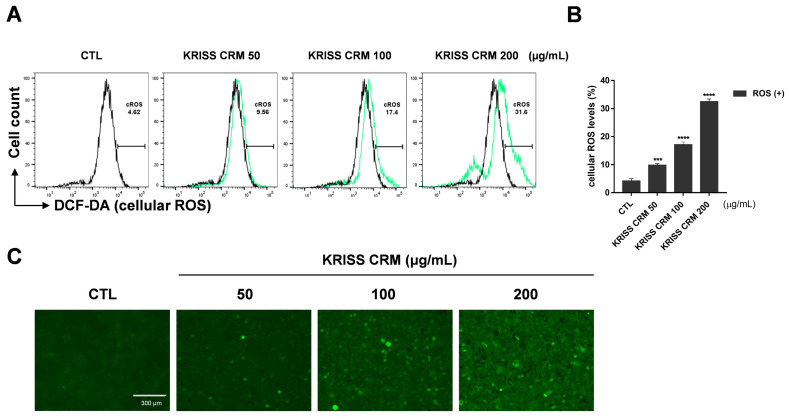
Cellular ROS were induced by KRISS CRM in BEAS-2B cells. (**A**) ROS production was analyzed via H_2_DCFDA staining-based (10 μM) flow cytometry. ROS levels were assessed by DCFH-DA after 48 h of exposure to 50 μg, 100 μg, and 200 μg of KRISS CRM in BEAS-2B cells. FACSVerse were used to measure the cellular ROS levels after the various concentrations (50, 100, and 200 μg/mL) of KRISS CRM treatment. It increased more than seven-fold in the 200 μg KRISS CRM treatment, and other CRM-treated samples also increased. (**B**) Quantification of ROS levels. Data are from individual experiments (*n* = 3) and are shown as mean fold change (FC) to control ± SEM. Statistical significance is shown as: *** *p* < 0.001, **** *p* ≤ 0.0001. (**C**) Fluorescence microscopy images of each sample well using the EVOS M5000 Green Fluorescent Protein (GFP) channel. Fluorescence image taken after 48 h of KRISS CRM treatment (scale bar: 300 μm).

To check whether KRISS CRM increased the imbalance of the redox state of a cell, we performed fluorescence-activated cell sorting (FACS) analysis using a ROS sensor dye, dichlorodihydrofluorescein diacetate (DCFH-DA). We investigated ROS levels after treatment with four doses of KRISS CRM. As expected, Figure 2C shows that cellular ROS levels increased after KRISS CRM treatment. Furthermore, according to flow cytometry analysis, BEAS-2B cells treated with KRISS CRM at doses of 100 μg to 200 μg significantly generated ROS at 17.4% to 31.6%, respectively, compared to 4.62% in the control sample, as shown in Figure 2A,B. Furthermore, we analyzed the increased intracellular ROS levels in BEAS-2B cells through a fluorescence microscope, the EVOS M5000 Imaging System.

### 3.3. KRISS CRM Increases Production of Mitochondrial Superoxide Anion Levels in Lung Cells

**Figure 3:**
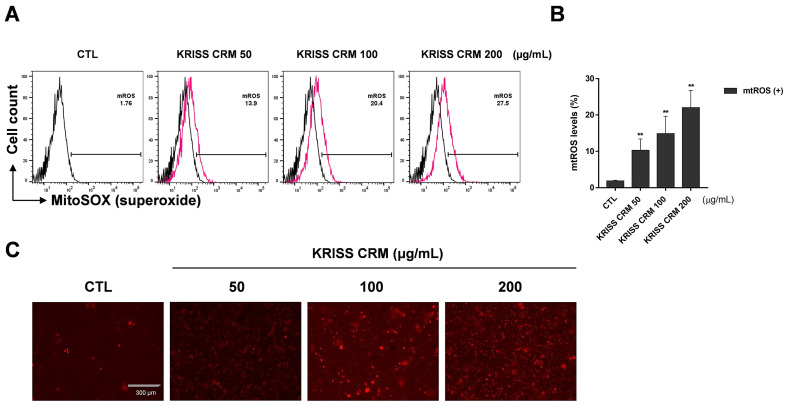
Mitochondrial superoxide levels were increased by KRISS CRM in BEAS-2B cells. (**A**) Mitochondrial superoxide anion levels were assessed using the MitoSOX red tracker after 48 h of exposure to 50 μg, 100 μg, and 200 μg of KRISS CRM in BEAS-2B cells. The 200 μg KRISS CRM-treated group increased compared to the untreated group in a dose-dependent manner. (**B**) Quantification of superoxide levels. Data are from individual experiments (*n* = 3) and are shown as mean fold change (FC) to control ± SEM. Statistical significance is shown as: ** *p* < 0.01. (**C**) Representative fluorescence images for each well using the Red Fluorescent Protein (RFP) channel. Fluorescence image taken after 48 h of KRISS CRM treatment (scale bar: 300 μm).

As the transition between oxidative stress and redox signaling is closely linked, we investigated the precise site of ROS generation, whether from cytosolic or mitochondrial production. We assessed the mitochondrial ROS levels using the specific probe for mitochondrial superoxide production, mitochondria-targeted triphenylphosphonium-linked hydroethidium (MitoSOX). For the flow cytometry analysis, we used the 5 μM working solution of MitoSOX with 0.5 M phosphate buffer at pH 7.4. As shown in Figure 3A,B, the KRISS CRM-treated groups increased mitochondrial superoxide anion levels in a dose-dependent manner. As shown in the microscopic analysis in Figure 3C, the intensity of RFP increased gradually in a dose-dependent manner. In the sample group, 50 μg, 100 μg, and 200 μg of KRISS CRM were treated for 48 h and harvested along with the untreated group (control). In the control group, superoxide levels were 1.76%, but after linear dose treatment with KRISS CRM, levels increased to 27.5%. We also checked the increased levels of superoxide using a fluorescence microscope.

### 3.4. KRISS CRM Decreases Mitochondrial Membrane Potential in Lung Cells

**Figure 4:**
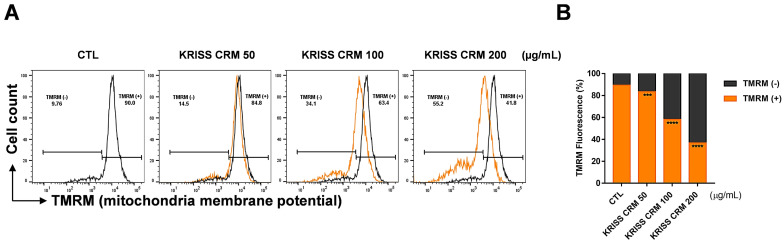
Production of the mitochondrial membrane potential was induced by KRISS CRM in human lung epithelial cells. (**A**) Mitochondrial membrane potential (MMP) was assessed by TMRM after 48 h of exposure to 50 μg, 100 μg, and 200 μg of KRISS CRM in BEAS-2B cells. Cells were then loaded with 10 μM TMRM and measured by flow cytometry and fluorescence microscopy. Untreated BEAS-2B cells were used as controls. (**B**) Quantification of MMP (∆ψm) levels. Data are from individual experiments (*n* = 3) and are shown as mean fold change (FC) to control ± SEM. Statistical significance is indicated as: *** *p* < 0.001, **** *p* ≤ 0.0001.

To demonstrate ∆ψm in BEAS-2B cells, TMRM, a lipophilic cation that accumulates in mitochondria in a membrane potential-dependent manner, was used. The human normal lung epithelial cell line was exposed to 50 μg to 200 μg of KRISS PM for 48 h and measured by flow cytometry analysis. As shown in Figure 4A,B, TMRM (+) denotes the intensity of TMRM, which decreased significantly to 41.8% at 200 μg, 63.4% at 100 μg, and 84.8% at 50 μg, in contrast to the control value of 90.0%. Thus, KRISS CRM may be the cause of BEAS-2B mitochondrial membrane depolarization and mitochondrial ROS.

### 3.5. KRISS CRM Increases Lipid ROS Levels in Human Lung Cells

**Figure 5:**
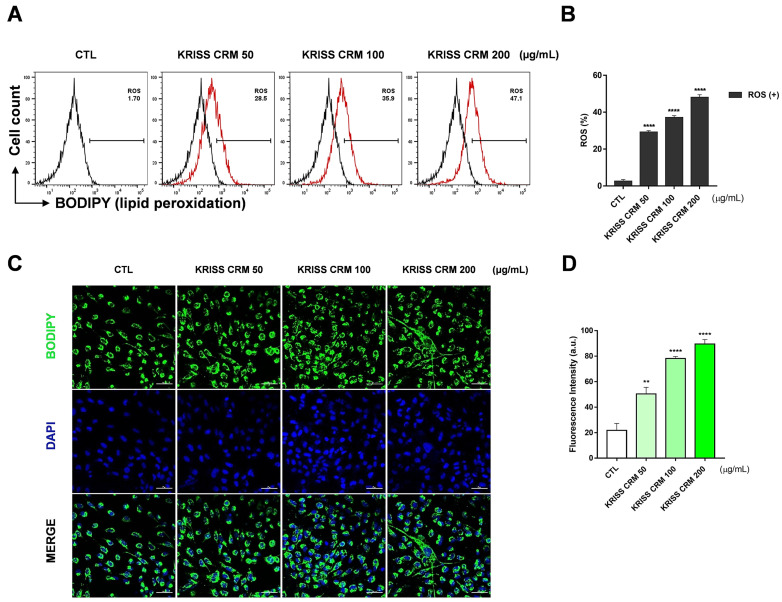
Lipid peroxidation was generated by KRISS CRM in BEAS-2B cells. (**A**) Lipid ROS levels were evaluated by C11-BODIPY^581/591^ after 48 h of exposure to 50 μg, 100 μg, and 200 μg of KRISS CRM in BEAS-2B cells. (**B**) Quantification of lipid ROS levels. Data are from individual experiments (*n* = 3) and are shown as mean fold change (FC) to control ± SEM. Statistical significance is indicated as: **** *p* ≤ 0.0001. (**C**) Confocal imaging of C11-BODIPY loaded cells: green, oxidized form of C11-BODIPY; blue, DAPI; merged images (green and blue channels). (**D**) Quantification of lipid peroxidation levels. Statistical significance is indicated as: ** *p* < 0.01, **** *p* ≤ 0.0001. Fluorescence image taken after 48 h of KRISS CRM treatment (scale bar: 50 μm).

We investigated the effects of KRISS CRM on lipid ROS generation using flow cytometry analysis with C11-BODIPY staining. The results indicated an increase in lipid peroxidation levels in BEAS-2B cells treated with concentrations of 50, 100, and 200 μg/mL of KRISS CRM, as shown in Figure 5. C11-BODIPY is a lipid peroxidation sensor, and the levels were increased to 28.5% at 50 μg, 35.9% at 100 μg, and 47.1% at 200 μg compared to the control value of 1.70%. Flow cytometry analysis confirmed that treatment with KRISS CRM significantly generated the lipid ROS levels in a dose-dependent manner, compared with untreated BEAS-2B cells as shown in Figure 5A,B. To determine the level of lipid peroxidation more visually, we used the lipid peroxidation fluorescence sensor C11-BODIPY for live cell image analysis (Figure 5C,D). The lipophilic fluorescent dye remains bound to the membrane, reacts with various ROS, and undergoes oxidation that shifts the emission peak from approximately 590 nm (red) to approximately 510 nm (green). Cells treated with KRISS CRM exhibited significantly greater emission of oxidized green fluorescence compared to untreated cells. These results show that KRISS CRM induces lipid ROS in human lung epithelial cells.

### 3.6. The Effect of KRISS CRM on the Expression Levels of mRNAs Involved in Oxidative Stress

**Figure 6:**
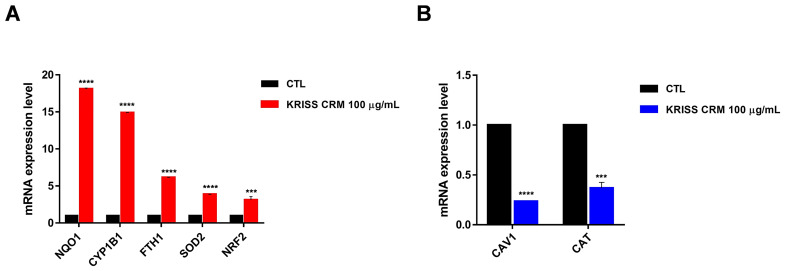
KRISS CRM affects mRNA expression levels of oxidative-stress-related genes. (**A**) Quantification of mRNA levels of NQO1, CYP1B1, FTH1, SOD2, and NRF was performed by RT-qPCR analysis. Statistical significance is indicated as: *** *p* < 0.001, **** *p* ≤ 0.0001. (**B**) Quantification of mRNA levels of CAV1 and CAT was performed by RT-qPCR. Statistical significance is indicated as: *** *p* < 0.001, **** *p* ≤ 0.0001.

To identify altered expression pathways and epigenetic changes that occur in response to KRISS CRM exposure in human lung epithelial cells, we used quantitative reverse transcription-polymerase chain reaction (RT-qPCR) techniques. We investigated whether KRISS CRM affects the expression levels of mRNAs associated with oxidative stress. RT-qPCR data showed that there was a significant increase in the mRNA levels of NQO1, CYP1B1, FTH1, SOD2, and NRF2 as shown in Figure 6A, suggesting the importance of the oxidative-stress-related pathway in human lung epithelial cells. Notably, NQO1, known for its protective role against oxidative stress and inhibition of ferroptosis, exhibited elevated expression levels. Conversely, CAV1, typically downregulated during oxidative stress, showed decreased expression levels in response to KRISS CRM exposure. These findings, shown in Figure 6A,B, confirm the induction of oxidative-stress-related genes at the mRNA level in human lung epithelial cells following exposure to KRISS CRM.

### 3.7. The Effect of KRISS CRM on the Expression Levels of mRNAs Involved in Ferroptosis

**Figure 7:**
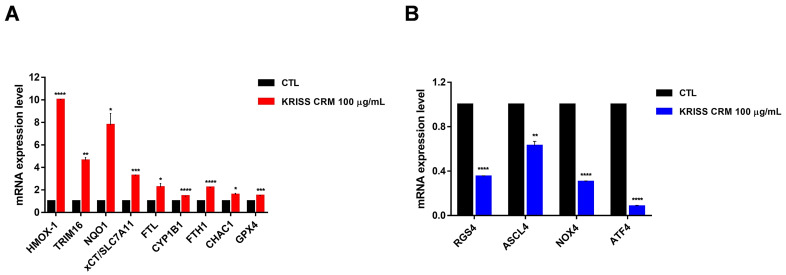
KRISS CRM affects mRNA expression levels of ferroptosis-related genes. (**A**) Quantification of mRNA levels of HMOX-1, TRIM16, NQO1, xCT/SLC7A11, FTL, CYP1B1, FTH1, CHAC1, and GPX4 was performed by RT-qPCR analysis. Statistical significance is indicated as: * *p* ≤ 0.05, ** *p* < 0.01, *** *p* < 0.001, **** *p* ≤ 0.0001. (**B**) Quantification of mRNA levels of RGS4, ASCL4, NOX4, and ATF4 was performed by RT-qPCR. Statistical significance is indicated as: ** *p* < 0.01, **** *p* ≤ 0.0001.

Interestingly, xCT/SLC7A11, SL7A11-AS1, TRIM16L, TRIM16, HMOX1, NQO1, FTL, CYP1B1, and one of the downregulated genes (RGS4) were known to be associated with the oxidative-stress-mediated ferroptosis pathway. Primers were designed to amplify several ferroptosis-related genes including xCT/SLC7A11, TRIM16, FTL, GPX4, CHAC1, and RGS4, which are known ferroptosis regulators. RT-qPCR data showed that there was a significant increase in the mRNA levels of xCT/SLC7A11, TRIM16, FTL, FTH1, CYP1B1, NQO1, HO-1, and GPX4 as shown in Figure 7A, suggesting significant modulation of the ferroptosis-related pathway in human lung epithelial cells. GPX4 (glutathione peroxidase 4), an antioxidant defense enzyme, is a key inhibitor of ferroptosis, which can repair oxidative damage to lipids. In addition, since RGS4 (regulator of G-protein signaling 4) is known to be downregulated during ferroptosis [47,48], we performed RT-qPCR and showed that RGS4 levels decreased upon the 100 µg of KRISS CRM treatment. As shown in the results, it was confirmed that RGS4, ASCL4, NOX4, and ATF4 levels decreased when treated with KRISS CRM (Figure 7B). On the other hand, nine ferroptosis-related genes were upregulated. According to our research, at the molecular level, KRISS CRM up- or downregulates several gene expressions that undergo ferroptosis.

### 3.8. KRISS CRM Affects Antioxidant Expression Levels in Lung Epithelial Cells

**Figure 8:**
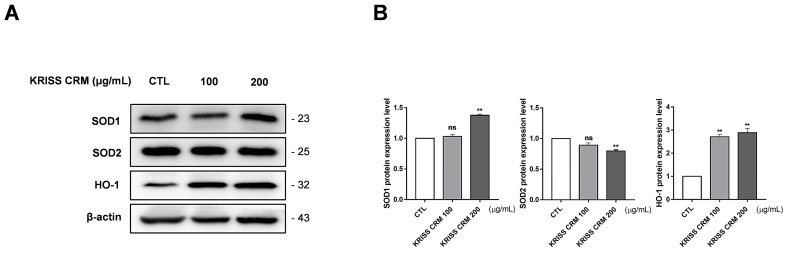
The level of antioxidant expression in lung epithelial cells altered by KRISS CRM. (**A**) Representative Western blots of SOD1, SOD2, and HO-1 in human lung epithelial cells treated with 100 μg and 200 μg of KRISS CRM for 48 h. (**B**) Relative SOD1, SOD2, and HO-1 protein expression in BEAS-2B cells after KRISS CRM treatment. Statistical significance is indicated as: ns = not significant, ** *p* < 0.01.

The protein expression levels of the antioxidant were confirmed by Western blot assay to find out if KRISS CRM affects the relationship between excessive ROS levels and antioxidants. We measured the expression of superoxide dismutase 1 (SOD1), superoxide dismutase 2 (SOD2), and heme oxygenase (HO-1) by Western blotting after treatment with 100 µg and 200 µg of KRISS CRM. KRISS CRM increased the SOD1 protein expression level and decreased the SOD2 protein expression level in a dose-dependent manner (Figure 8A,B). On the other hand, the expression of HO-1 was significantly increased after treatment with 100 µg and 200 µg KRISS CRM compared to the untreated group. We confirmed that after 48 h of treatment with KRISS CRM, antioxidant biomolecular changes occur. Therefore, excessive ROS levels induced by KRISS CRM can activate the antioxidant defense system.

### 3.9. KRISS CRM Exposure Induces Ferroptosis-Related Protein Changes and Iron Accumulation in Human Lung Cells

**Figure 9:**
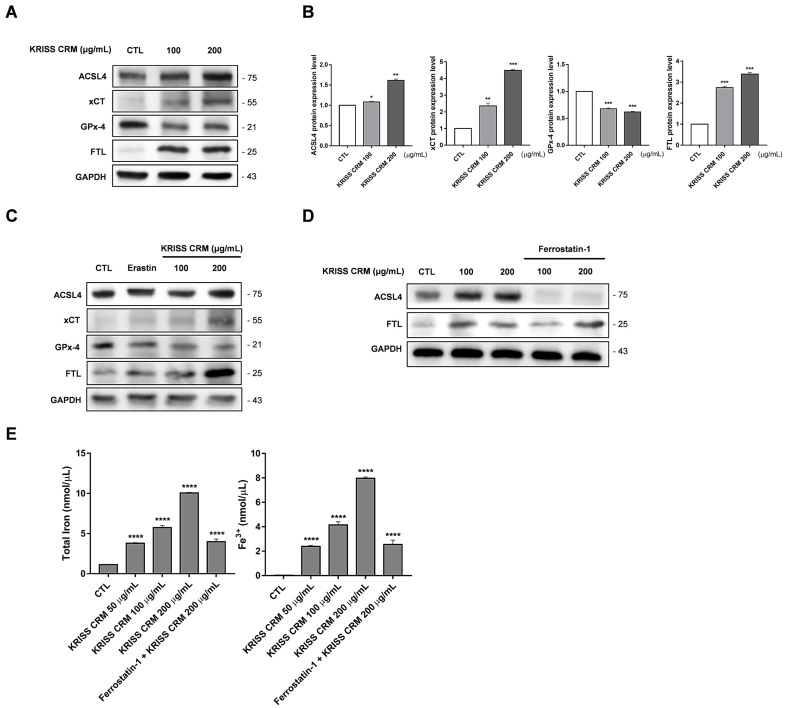
Induction of ferroptosis-related protein alterations and iron accumulation in human lung cells by KRISS CRM exposure. (**A**) Representative Western blots of ACSL4, xCT, GPx-4, and FTL in human lung epithelial cells treated with 100 μg and 200 μg of KRISS CRM for 48 h. Protein expression levels were normalized to GAPDH. (**B**) Relative ACSL4, xCT, GPx-4, and FTL protein expression in BEAS-2B cells after KRISS CRM treatment. Statistical significance is indicated as: * *p* ≤ 0.05, ** *p* < 0.01, *** *p* < 0.001. (**C**) Western blot analysis of ACSL4, xCT, GPx4, and FTL in lung cells treated with Erastin (1 μM) and 100 μg and 200 μg of KRISS CRM for 48 h. (**D**) The effect of Ferrostatin-1 on ACSL4 and FTL protein expression was assessed by Western blot analysis. Lung cells were pretreated with Ferrostatin-1 (12 μM) before exposure to KRISS CRM. (**E**) Total iron and Fe^3+^ levels were measured in BEAS-2B cells after treatment with 100 μg and 200 μg of KRISS CRM, as well as pretreatment with Ferrostatin-1 (12 μM) prior to treatment with KRISS CRM at 200 μg. Statistical significance is indicated as: **** *p* ≤ 0.0001.

To investigate the effects of KRISS CRM exposure on ferroptosis-related proteins and iron accumulation, we conducted Western blot and iron assay analyses. Western blot analysis revealed significant alterations in the expression levels of ferroptosis-related proteins following KRISS CRM exposure. Specifically, ACSL4, xCT, and FTL exhibited increased expression, while GPx-4 showed decreased expression, compromising cellular antioxidant defense (Figure 9A,B). Additionally, treatment with Erastin and 100 μg and 200 μg concentrations of KRISS CRM resulted in changes in the expression levels of ACSL4, xCT, GPx-4, and FTL (Figure 9C). Pretreatment with Ferrostatin-1 followed by KRISS CRM treatment further confirmed alterations in ferroptosis-related protein expression, validating our findings (Figure 9D). Iron accumulation was observed in human lung cells exposed to KRISS CRM, as indicated by increased total iron and Fe^3+^ levels (Figure 9E). Treatment with Ferrostatin-1 resulted in a reduction in iron levels, suggesting that iron accumulation occurs in response to particulate matter exposure. These results provide valuable insights into the mechanisms underlying the cellular response to particulate matter exposure and offer potential targets for therapeutic intervention in oxidative-stress-related diseases.

## 4. Discussion

Air pollution is highly toxic to the human respiratory system and causes cell death by apoptosis, necrosis, autophagy, and ferroptosis [49]. However, little is known about their precise effects on lung health because the particles vary in size, concentration, composition, and duration of exposure. Prolonged exposure to certain types of particulate matter, especially fine particles (PM2.5), has been linked to an increased risk of lung cancer [50]. In addition, research into the effects of PM10 on lung health underlines the complexity of the effects of particulate matter on respiratory well-being [51,52]. This connection is based on the potential of particles to carry carcinogens and cause gradual damage to lung tissue over time [53]. Just as differences in the size of PM particles affect lung health, the composition of PM particles produced by vehicle emissions, industrial processes, construction activities, and natural sources such as pollen and dust is also important. Particulate matter is used as a key indicator of ambient air quality and to measure its potential impact on human health and the environment [54]. Significant research efforts are underway to improve the composition and properties of particulate matter, providing the basis for accurate and reliable air quality measurements.

The results of current studies on ferroptosis after exposure to PM are relatively poorly known, and current prevention strategies are also inadequate [55]. In the current study, we focused on the new certified reference material for particulate matter developed by the Korea Research Institute of Standards and Science (KRISS), and used it to demonstrate the cytotoxic effects of this material in human lung epithelial cells via the oxidative-stress-mediated ferroptosis pathway. Ferroptosis is characterized by the accumulation of lipid peroxides and iron-dependent reactive oxygen species (ROS), leading to oxidative damage and, ultimately, cell death [56]. In this study, we report that KRISS CRM induces ferroptosis along with excessive ROS generation, lipid peroxidation, iron accumulation, GPX4 inhibition, and mitochondrial damage.

We conducted our study using BEAS-2B cells, well-established immortalized human bronchial epithelial cells that represent normal lung cells, providing insights into cytotoxic effects relevant to lung physiology [57,58,59]. Compared to lung cancer cell lines such as A549 or H1437, BEAS-2B cells exhibit greater genetic stability and are less prone to genomic alterations, making them a more reliable platform for studying cytotoxic responses [60,61]. Additionally, as non-tumorigenic cells, BEAS-2B cells better mimic the in vivo microenvironment, enhancing the translatability of our findings to physiological settings [62]. Therefore, we selected BEAS-2B cells for our cytotoxicity study due to their suitability as a model system to investigate cellular responses closely resembling normal lung tissue, thereby ensuring the scientific rigor and relevance of our research. 

For the KRISS CRM positive control, NIST SRM 1648a, established by the National Institute of Standards and Technology (NIST), serves as a certified reference material widely utilized for assessing contaminants such as particulate matter in environmental and biological research [63,64,65]. We conducted MTS assays using NIST SRM 1648a as a positive control for the KRISS CRM, a certified reference material for fine particulate matter. The results demonstrated comparable cell viability between NIST SRM 1648a and KRISS CRM, affirming the utility of the latter in our study (Supplementary Figure S1). This observation underscores the reliability and validity of our experimental approach, providing valuable insights into the biological responses elicited by KRISS CRM exposure. We also conducted FACS experiments using NIST SRM as the positive control, alongside MTS assays. We utilized NIST SRM 1648a, a widely recognized standard material in particulate matter research, as a positive control to assess the H_2_DCFDA levels in comparison to KRISS CRM, a certified reference material for fine particulate matter. The results revealed that both NIST SRM 1648a and KRISS CRM exhibited an increase in H_2_DCFDA levels corresponding to the concentration gradient. This observation underscores the consistency of the H_2_DCFDA response to varying concentrations of particulate matter across different reference materials, highlighting the reliability of our experimental approach. The Supplementary Figure S2 provides valuable insights into the H_2_DCFDA response induced by KRISS CRM and NIST SRM 1648a exposure. Through these assays, we confirmed that KRISS CRM exhibits similar effects in the biological environment as NIST SRM 1648a. However, our focus remains on elucidating the cytotoxicity of KRISS CRM, which represents a novel development as a potential first reference material for particulate matter in Korea. Given the current absence of a clear reference material for Korean particulate matter, assessing its environmental indicators and potential harmful effects on the human body poses significant challenges. Therefore, this study aims to shed light on the cytotoxic effects associated with KRISS CRM and elucidate the mechanisms underlying diseases caused by exposure to particulate matter. 

In numerous studies to date, research on the environmental and health impacts has predominantly focused on fine dust particles with a diameter of 2.5 μM or less, known as PM2.5, and particulate matter with a diameter of 10 μM or less, denoted as PM10. This study utilized the fine dust standard material KRISS CRM with a particle size of 20 μM or less, covering both PM2.5 and PM10. Therefore, accurately discerning the toxicity associated with a specific size of fine dust remains challenging. However, as the elemental composition of KRISS CRM has been identified through inorganic analysis, a precise investigation into the toxicity attributed to its constituents is feasible. While this study confirmed toxicity levels, future research could delve into determining the toxicity related to specific elemental components. A new approach is needed to address the growing environmental challenges posed by increasing chemical pollutants. The concept of the “exposome” aims to comprehensively analyze an individual’s lifetime exposure to environmental factors. By exploring the exposome, researchers aim to unravel the complex relationship between environmental exposures and health, potentially providing new insights into disease causation and prevention, and personalized therapeutic strategies [66,67,68].

Reactive oxygen species (ROS) are compounds containing oxygen formed as natural byproducts of normal cellular metabolism, known as critical molecules in processes such as cell cycle progression, differentiation, and normal cell death [69]. However, the overproduction of ROS is the main cause of oxidative stress, typically defined as an imbalance that promotes ROS generation over antioxidant defenses [70]. Additionally, ROS play important roles in physiological processes and cellular signaling, and are also associated with ferroptosis [71]. A previous study reported that PM2.5 exposure induces the elevation of cellular ROS, and this excessive ROS level may lead to the production of highly reactive lipid radicals that drive oxidative stress [72]. Consistent with this, we found that exposure to KRISS CRM increased the cellular and mitochondrial ROS levels and altered the expression of antioxidants, which are inhibitors of excessive ROS. Maintaining the delicate balance of ROS levels is critical to maintaining cellular homeostasis and a healthy respiratory system [73].

The majority of reactive oxygen species (ROS) are generated during the mitochondrial oxidative phosphorylation process, closely linking ROS with mitochondrial activity [74,75]. This correlation is particularly associated with cellular oxidative stress, where excessive ROS production can impair mitochondrial function, disrupting cellular balance [76]. Conversely, mitochondrial dysfunction can exacerbate ROS generation, leading to increased oxidative stress within the cell [77]. In our study, we assessed the effects of KRISS CRM on mitochondrial membrane potential using tetramethylrhodamine methyl ester (TMRM). However, TMRM primarily measures fluorescence related to the electrochemical gradient within mitochondria, limiting its direct measurement of ROS [78]. To complement this, we conducted additional ROS detection experiments using MitoSOX, a specific fluorescent dye designed for detecting mitochondrial ROS.

In addition to mitochondria and NADPH oxidases, other sources of reactive oxygen species (ROS) could be induced by PM. While nitric oxide (NO) produced by nitric oxide synthase (NOS) is beneficial, excessive production or uncoupling of NOS can lead to the generation of ROS, particularly superoxide anion [79]. Reactive oxygen species can also be generated through the process of lipid peroxidation, wherein free radicals attack lipid molecules, resulting in the production of lipid hydroperoxides and other reactive species [80,81]. ROS can also be produced during inflammatory responses, through the action of certain enzymes like myeloperoxidase and lipoxygenases, and as byproducts of cellular metabolism [82]. Lipid peroxidation induced by reactive oxygen species (ROS) plays a crucial role in various cell death processes, including apoptosis, autophagy, and ferroptosis [83]. Ferroptosis, a distinct mode of cell death, is characterized by iron-dependent cell demise resulting from severe membrane damage caused by lipid peroxidation [79,84]. In our study, we employed BODIPY for ROS detection to gain insights into lipid peroxidation. Our results demonstrated that KRISS CRM induced ROS production, leading to lipid peroxidation. However, it is essential to acknowledge the limitations inherent in our experimental approach and data interpretation. Future research should further investigate the mechanistic insights provided by BODIPY-mediated ROS detection and its relevance to lipid-peroxidation-related pathologies. Ultimately, our findings emphasize the importance of utilizing sensitive probes like BODIPY to unravel complex oxidative processes and their pathological implications.

ROS play a biologically important role in many physiological systems, including immunity, differentiation, proliferation, autophagy regulation, and hypoxia tolerance. They control a wide range of signaling pathways through direct interactions with proteins and modulation of transcription factors and gene expression [85,86]. However, excessive ROS levels can be detrimental, causing damage to cellular components such as DNA, RNA, proteins, and lipids [87]. To counteract these effects, cells activate defense mechanisms including the NRF2 pathway, glutathione peroxidase (GPx), thioredoxin (Trx), catalase (CAT), superoxide dismutase (SOD), and other antioxidant ROS scavengers [88]. Failure to regulate ROS levels appropriately can lead to cell death, emphasizing the importance of understanding the delicate balance between ROS generation and antioxidant defense mechanisms [89]. Our study focuses on the consequences of dysregulated ROS levels, particularly the activation of ferroptosis signaling pathways, highlighting the need to develop strategies to combat oxidative-stress-related diseases and improve overall health.

The experiment was conducted regarding DCFH-DA being the indicator of directly measuring the redox state of a cell in a previous experiment [90,91,92]. Detecting ROS in biological systems poses challenges due to method sensitivity and probe specificity. Treating ROS, antioxidants, and oxidative damage as unified concepts can hinder the precise interpretation of experiments, thereby overlooking the necessity of establishing specific molecular mechanisms. Therefore, in order to adhere to these principles, it is imperative to measure the effects of specific ROS and/or oxidative products, as well as antioxidants [93]. In this study, we measured the degree of DCFH-DA oxidation as an indicator of the cellular redox state, closely linked to reactive oxygen species (ROS). In normal cellular function, the redox state maintains a balance, but excessive generation of ROS or an imbalance in the redox state can contribute to cellular damage and disease onset. The redox state of cells naturally regulates and controls the generation of ROS, which can increase due to various factors, thereby minimizing cellular damage and protecting the cells [94,95]. Thus, 2′-7′-dichlorodihydrofluorescein diacetate (DCFH-DA), a small-molecule fluorescent probe, is commonly utilized to assess ROS levels within cells, providing a straightforward technique for monitoring ROS changes over time [90]. Therefore, we measured the change in DCF levels after treatment with KRISS CRM, a reference material of particulate matter, to identify changes in total cellular ROS levels due to redox state imbalance in cells.

Nuclear factor erythroid-2-related factor 2 (Nrf2) regulates antioxidant responses, protecting cells from oxidative stress and related pathologies, whereas reactive oxygen species (ROS) induce cellular damage [96,97]. The effective activation of Nrf2 requires proper nuclear translocation [98]. To determine this, it is imperative to perform a nuclear translocation assay to verify efficient translocation of Nrf2 into the nucleus. These experiments play a key role in understanding the signaling pathways associated with Nrf2 nuclear translocation [99]. In addition, such studies are expected to contribute significantly to the understanding of the mechanisms behind critical physiological responses such as antioxidant activation. Nrf2 is activated under oxidative stress conditions, inducing the expression of antioxidant response genes such as HO-1, which reduces oxidative stress by promoting heme oxidation and glutathione synthesis [100,101]. GPx-4 plays a crucial role in protecting cell membranes from lipid peroxidation, thereby collectively suppressing ferroptosis. Exposure to fine dust, like particulate matter, is known to induce oxidative stress, leading to the activation of Nrf2 and subsequent upregulation of HO-1 [102,103]. However, exposure to fine dust can also decrease GPx-4 expression, potentially enhancing lipid peroxidation [104]. In this study, we investigated the impact of exposure to KRISS CRM on the Nrf2-HO-1-GPx-4 pathway. Supplementary Figure S3 reveals an increase in Keap1 expression, indicating a notable shift in the cell’s response to oxidative stress. This upregulation of Keap1 suggests a potential suppression of antioxidant response gene expression, highlighting a compromised cellular defense mechanism against oxidative damage [104]. Conversely, as shown in Figure 8A, the increase in HO-1 may represent an attempt by the cell to counteract oxidative stress [105,106]. However, our findings in Figure 9A suggest that the decrease in GPx-4 expression suggests a compromised ability to inhibit lipid peroxidation in cell membranes, a key mechanism in ferroptosis. These protein alterations may thus provide insights into the mechanisms involved in ferroptosis signaling pathways.

ACSL4 is one of the key genes associated with ferroptosis, regulating this process by altering the composition of cellular membrane lipids. Activation of ACSL4 increases levels of unsaturated fatty acids within cells, which promotes lipid peroxidation and consequently induces ferroptosis [107,108]. On the other hand, xCT is a cystine-glutamate transporter responsible for exchanging cystine within cells [109]. This transporter plays a crucial role in providing cysteine for glutachione synthesis, which is essential for alleviating oxidative stress and detoxifying harmful substances within cells [110,111]. Consequently, impairment of xCT function leads to decreased glutachione levels, exposing cells to oxidative stress and potentially inducing ferroptosis [112,113]. Thus, xCT serves as a marker for ferroptosis because its activity level reflects the sensitivity of cells to ferroptosis induction. Typically, decreased expression of xCT leads to a reduction in intracellular cystine levels, resulting in diminished glutathione synthesis and rendering cells more vulnerable to oxidative stress and ferroptosis induction [114,115]. Conversely, reduced GPx-4, a glutathione peroxidase enzyme, results in increased lipid peroxidation in cell membranes, potentially triggering ferroptosis [116,117]. FTL, known as the ferritin light chain, is part of ferritin, a protein essential for storing and regulating iron. Ferritin prevents iron accumulation in cells and maintains it in a stable state, protecting against oxidative stress and iron toxicity [118]. Inhibiting FTL reduces ferritin’s ability to store iron in a stable form, increasing the risk of iron toxicity and oxidative stress in cells [37,119]. Therefore, Figure 9A demonstrates the regulation of ACSL4, xCT, GPx-4, and FTL upon treatment with KRISS CRM, indicating changes in the expression levels of key proteins associated with ferroptosis.

In daily life, humans are exposed to various particulate matter. Prolonged or short-term exposure to high concentrations of such particles is associated with a range of respiratory and cardiovascular diseases [120,121]. Our observation indicates that KRISS CRM induces intracellular and mitochondrial reactive oxygen species (ROS), ultimately leading to cellular apoptosis. The precise mechanisms underlying cell death caused by particulate matter are not yet fully elucidated, presenting an ongoing challenge for many researchers. However, in this study, we confirmed that excessive lipid peroxidation occurs due to elevated levels of ROS, resulting in subsequent cell death. This suggests a potential avenue for further investigation into ferroptosis, a form of iron-dependent cell death. In addition to ferroptosis, which is the focus of our research, cell death induced by particulate matter is diverse in many studies, predominantly involving apoptosis, autophagy, and necrosis [122,123,124]. Therefore, further studies on diverse forms of cell death would be an interesting avenue for future research.

## 5. Conclusions

In our research, we examined the potential cytotoxic impact of exposure to KRISS CRM on human lung epithelial cells. Our investigation revealed that KRISS CRM induces the production of various reactive oxygen species (ROS), including cellular, mitochondrial, and lipid ROS, and in particular, excessive ROS generation. Additionally, we observed that this excessive ROS production induces oxidative stress through various physiological pathways, particularly those associated with ferroptosis regulators and antioxidants such as xCT/SLC7A11, TRIM16, FTL, FTH1, CYP1B1, NQO1, HO-1, GPX4, SOD1, and SOD2. Therefore, based on these findings, KRISS CRM 109-02-004 can be considered a practical tool for assessing the impact of environmental indicators. Furthermore, it holds potential for elucidating the underlying mechanisms through which it may exert adverse effects on human health, serving as a valuable tool for future investigations.

## Data Availability

The data presented in this study are available in the article.

## Supplementary Materials

The following supporting information can be downloaded at https://www.mdpi.com/article/10.3390/toxics12020161/s1, Figure S1: The cell viability of KRISS CRM 109-02-004 and NIST SRM 1648a; Figure S2: Cellular ROS were induced by NIST SRM in BEAS-2B cells; Figure S3: The mRNA expression level of Keap1; Table S1: Certified values of KRISS CRM 109-02-004, urban particulate matter; Table S2: Approximate contents of elements in KRISS CRM 109-02-004, urban particulate matter; Table S3: The primer sequence of genes used in RT-qPCR. References [125,126,127,128] are cited in the Supplementary Materials.

## References

[1] Schraufnagel D.E. (2020). The health effects of ultrafine particles. Exp. Mol. Med..

[2] Shehab M.A., Pope F.D. (2019). Effects of short-term exposure to particulate matter air pollution on cognitive performance. Sci. Rep..

[3] Raaschou-Nielsen O., Beelen R., Wang M., Hoek G., Andersen Z.J., Hoffmann B., Stafoggia M., Samoli E., Weinmayr G., Dimakopoulou K. (2016). Particulate matter air pollution components and risk for lung cancer. Environ. Int..

[4] Xing Y.-F., Xu Y.-H., Shi M.-H., Lian Y.-X. (2016). The impact of PM2.5 on the human respiratory system. J. Thorac. Dis..

[5] Thangavel P., Park D., Lee Y.-C. (2022). Recent insights into particulate matter (PM2.5)-mediated toxicity in humans: An overview. Int. J. Environ. Res. Public Health.

[6] Park M., Joo H.S., Lee K., Jang M., Kim S.D., Kim I., Borlaza L.J.S., Lim H., Shin H., Chung K.H. (2018). Differential toxicities of fine particulate matters from various sources. Sci. Rep..

[7] Dumax-Vorzet A.F., Tate M., Walmsley R., Elder R.H., Povey A.C. (2015). Cytotoxicity and genotoxicity of urban particulate matter in mammalian cells. Mutagenesis.

[8] Riva D., Magalhães C.B., Lopes A., Lanças T., Mauad T., Malm O., Valença S.S., Saldiva P.H., Faffe D.S., Zin W.A. (2011). Low dose of fine particulate matter (PM2.5) can induce acute oxidative stress, inflammation and pulmonary impairment in healthy mice. Inhal. Toxicol..

[9] Wu W., Jin Y., Carlsten C. (2018). Inflammatory health effects of indoor and outdoor particulate matter. J. Allergy Clin. Immunol..

[10] Dixon S.J., Lemberg K.M., Lamprecht M.R., Skouta R., Zaitsev E.M., Gleason C.E., Patel D.N., Bauer A.J., Cantley A.M., Yang W.S. (2012). Ferroptosis: An iron-dependent form of nonapoptotic cell death. Cell.

[11] Gao M., Yi J., Zhu J., Minikes A.M., Monian P., Thompson C.B., Jiang X. (2019). Role of mitochondria in ferroptosis. Mol. Cell.

[12] Dixon S.J. (2017). Ferroptosis: Bug or feature?. Immunol. Rev..

[13] Do Van B., Gouel F., Jonneaux A., Timmerman K., Gelé P., Pétrault M., Bastide M., Laloux C., Moreau C., Bordet R. (2016). Ferroptosis, a newly characterized form of cell death in Parkinson’s disease that is regulated by PKC. Neurobiol. Dis..

[14] Du J., Zhou Y., Li Y., Xia J., Chen Y., Chen S., Wang X., Sun W., Wang T., Ren X. (2020). Identification of Frataxin as a regulator of ferroptosis. Redox Biol..

[15] Liang N.-N., Zhao Y., Guo Y.-Y., Zhang Z.-H., Gao L., Yu D.-X., Xu D.-X., Xu S. (2022). Mitochondria-derived reactive oxygen species are involved in renal cell ferroptosis during lipopolysaccharide-induced acute kidney injury. Int. Immunopharmacol..

[16] Gao M., Monian P., Pan Q., Zhang W., Xiang J., Jiang X. (2016). Ferroptosis is an autophagic cell death process. Cell Res..

[17] Ren J.-X., Li C., Yan X.-L., Qu Y., Yang Y., Guo Z.-N. (2021). Crosstalk between oxidative stress and ferroptosis/oxytosis in ischemic stroke: Possible targets and molecular mechanisms. Oxid. Med. Cell. Longev..

[18] Kuang F., Liu J., Tang D., Kang R. (2020). Oxidative damage and antioxidant defense in ferroptosis. Front. Cell Dev. Biol..

[19] Cheng Y., Song Y., Chen H., Li Q., Gao Y., Lu G., Luo C. (2021). Ferroptosis mediated by lipid reactive oxygen species: A possible causal link of neuroinflammation to neurological disorders. Oxid. Med. Cell. Longev..

[20] Forcina G.C., Dixon S.J. (2019). GPX4 at the crossroads of lipid homeostasis and ferroptosis. Proteomics.

[21] Liang H., Ran Q., Jang Y.C., Holstein D., Lechleiter J., McDonald-Marsh T., Musatov A., Song W., Van Remmen H., Richardson A. (2009). Glutathione peroxidase 4 differentially regulates the release of apoptogenic proteins from mitochondria. Free Radic. Biol. Med..

[22] Liu J., Kang R., Tang D. (2022). Signaling pathways and defense mechanisms of ferroptosis. FEBS J..

[23] Ighodaro O.M., Akinloye O.A. (2018). First line defence antioxidants-superoxide dismutase (SOD), catalase (CAT) and glutathione peroxidase (GPX): Their fundamental role in the entire antioxidant defence grid. Alex. J. Med..

[24] Ma Q. (2013). Role of nrf2 in oxidative stress and toxicity. Annu. Rev. Pharmacol. Toxicol..

[25] Xiang Q., Zhao Y., Lin J., Jiang S., Li W. (2022). The Nrf2 antioxidant defense system in intervertebral disc degeneration: Molecular insights. Exp. Mol. Med..

[26] Zhao T., Yu Z., Zhou L., Wang X., Hui Y., Mao L., Fan X., Wang B., Zhao X., Sun C. (2022). Regulating Nrf2-GPx4 axis by bicyclol can prevent ferroptosis in carbon tetrachloride-induced acute liver injury in mice. Cell Death Discov..

[27] Shin D., Kim E.H., Lee J., Roh J.-L. (2018). Nrf2 inhibition reverses resistance to GPX4 inhibitor-induced ferroptosis in head and neck cancer. Free Radic. Biol. Med..

[28] Dworzański J., Strycharz-Dudziak M., Kliszczewska E., Kiełczykowska M., Dworzańska A., Drop B., Polz-Dacewicz M. (2020). Glutathione peroxidase (GPx) and superoxide dismutase (SOD) activity in patients with diabetes mellitus type 2 infected with Epstein-Barr virus. PLoS ONE.

[29] Sato T., Seyama K., Sato Y., Mori H., Souma S., Akiyoshi T., Kodama Y., Mori T., Goto S., Takahashi K. (2006). Senescence marker protein-30 protects mice lungs from oxidative stress, aging, and smoking. Am. J. Respir. Crit. Care Med..

[30] Lee N., Carlisle A.E., Peppers A., Park S.J., Doshi M.B., Spears M.E., Kim D. (2021). xCT-driven expression of GPX4 determines sensitivity of breast cancer cells to ferroptosis inducers. Antioxidants.

[31] Vomund S., Schäfer A., Parnham M.J., Brüne B., Von Knethen A. (2017). Nrf2, the master regulator of anti-oxidative responses. Int. J. Mol. Sci..

[32] Soares M.P., Hamza I. (2016). Macrophages and iron metabolism. Immunity.

[33] Chiang S.-K., Chen S.-E., Chang L.-C. (2018). A dual role of heme oxygenase-1 in cancer cells. Int. J. Mol. Sci..

[34] Wu D., Hu Q., Wang Y., Jin M., Tao Z., Wan J. (2022). Identification of HMOX1 as a critical ferroptosis-related gene in atherosclerosis. Front. Cardiovasc. Med..

[35] Liu J., Ren Z., Yang L., Zhu L., Li Y., Bie C., Liu H., Ji Y., Chen D., Zhu M. (2022). The NSUN5-FTH1/FTL pathway mediates ferroptosis in bone marrow-derived mesenchymal stem cells. Cell Death Discov..

[36] He J., Abikoye A.M., McLaughlin B.P., Middleton R.S., Sheldon R., Jones R.G., Schafer Z.T. (2023). Reprogramming of iron metabolism confers ferroptosis resistance in ECM-detached cells. Iscience.

[37] Mou Y., Wang J., Wu J., He D., Zhang C., Duan C., Li B. (2019). Ferroptosis, a new form of cell death: Opportunities and challenges in cancer. J. Hematol. Oncol..

[38] Sun Y., Chen P., Zhai B., Zhang M., Xiang Y., Fang J., Xu S., Gao Y., Chen X., Sui X. (2020). The emerging role of ferroptosis in inflammation. Biomed. Pharmacother..

[39] Battaglia A.M., Chirillo R., Aversa I., Sacco A., Costanzo F., Biamonte F. (2020). Ferroptosis and cancer: Mitochondria meet the “iron maiden” cell death. Cells.

[40] Chen G.-H., Song C.-C., Pantopoulos K., Wei X.-L., Zheng H., Luo Z. (2022). Mitochondrial oxidative stress mediated Fe-induced ferroptosis via the NRF2-ARE pathway. Free Radic. Biol. Med..

[41] Wagner P.D. (2015). The physiological basis of pulmonary gas exchange: Implications for clinical interpretation of arterial blood gases. Eur. Respir. J..

[42] Siobal M.S. (2016). Monitoring exhaled carbon dioxide. Respir. Care.

[43] Yang L., Li C., Tang X. (2020). The impact of PM2. 5 on the host defense of respiratory system. Front. Cell Dev. Biol..

[44] Leikauf G.D., Kim S.-H., Jang A.-S. (2020). Mechanisms of ultrafine particle-induced respiratory health effects. Exp. Mol. Med..

[45] Engels S.M., Kamat P., Pafilis G.S., Li Y., Agrawal A., Haller D.J., Phillip J.M., Contreras L.M. (2023). Particulate matter composition drives differential molecular and morphological responses in lung epithelial cells. PNAS Nexus.

[46] Zhang R., Liu C., Zhou G., Sun J., Liu N., Hsu P.-C., Wang H., Qiu Y., Zhao J., Wu T. (2018). Morphology and property investigation of primary particulate matter particles from different sources. Nano Res..

[47] Stockwell B.R. (2022). Ferroptosis turns 10: Emerging mechanisms, physiological functions, and therapeutic applications. Cell.

[48] Jian X., Zhao G., Chen H., Wang Y., Li J., Xie L., Li B. (2022). Revealing a novel contributing landscape of ferroptosis-related genes in Parkinson’s disease. Comput. Struct. Biotechnol. J..

[49] Andreau K., Leroux M., Bouharrour A. (2012). Health and cellular impacts of air pollutants: From cytoprotection to cytotoxicity. Biochem. Res. Int..

[50] Liu C., Yang D., Liu Y., Piao H., Zhang T., Li X., Zhao E., Zhang D., Zheng Y., Tang X. (2023). The effect of ambient PM2.5 exposure on survival of lung cancer patients after lobectomy. Environ. Health.

[51] Kwon S.O., Hong S.H., Han Y.-J., Bak S.H., Kim J., Lee M.K., London S.J., Kim W.J., Kim S.-Y. (2020). Long-term exposure to PM10 and NO_2_ in relation to lung function and imaging phenotypes in a COPD cohort. Respir. Res..

[52] Mebrahtu T.F., Santorelli G., Yang T.C., Wright J., Tate J., McEachan R.R.C. (2023). The effects of exposure to NO_2_, PM2.5 and PM10 on health service attendances with respiratory illnesses: A time-series analysis. Environ. Pollut..

[53] Sharma D., Jain S. (2020). Carcinogenic risk from exposure to PM2.5 bound polycyclic aromatic hydrocarbons in rural settings. Ecotoxicol. Environ. Saf..

[54] Dong J., Wang Y., Wang L., Zhao W., Huang C. (2022). Assessment of PM2.5 exposure risk towards SDG indicator 11.6. 2–A case study in Beijing. Sustain. Cities Soc..

[55] Johnson N.M., Hoffmann A.R., Behlen J.C., Lau C., Pendleton D., Harvey N., Shore R., Li Y., Chen J., Tian Y. (2021). Air pollution and children’s health—A review of adverse effects associated with prenatal exposure from fine to ultrafine particulate matter. Environ. Health Prev. Med..

[56] Yang W.S., Stockwell B.R. (2016). Ferroptosis: Death by lipid peroxidation. Trends Cell Biol..

[57] Abbas I., Badran G., Verdin A., Ledoux F., Roumie M., Lo Guidice J.-M., Courcot D., Garçon G. (2019). In vitro evaluation of organic extractable matter from ambient PM2.5 using human bronchial epithelial BEAS-2B cells: Cytotoxicity, oxidative stress, pro-inflammatory response, genotoxicity, and cell cycle deregulation. Environ. Res..

[58] Podder B., Song H.-Y., Kim Y.-S. (2014). Naringenin Exerts Cytoprotective Effect Against Paraquat-Induced Toxicity in Human Bronchial Epithelial BEAS-2B Cells Through NRF2 Activation. J. Microbiol. Biotechnol..

[59] Veranth J.M., Reilly C.A., Veranth M.M., Moss T.A., Langelier C.R., Lanza D.L., Yost G.S. (2004). Inflammatory cytokines and cell death in BEAS-2B lung cells treated with soil dust, lipopolysaccharide, and surface-modified particles. Toxicol. Sci..

[60] Biola-Clier M., Beal D., Caillat S., Libert S., Armand L., Herlin-Boime N., Sauvaigo S., Douki T., Carriere M. (2016). Comparison of the DNA damage response in BEAS-2B and A549 cells exposed to titanium dioxide nanoparticles. Mutagenesis.

[61] Garcia-Canton C., Minet E., Anadon A., Meredith C. (2013). Metabolic characterization of cell systems used in in vitro toxicology testing: Lung cell system BEAS-2B as a working example. Toxicol. Vitr..

[62] Phan T.H., Shi H., Denes C.E., Cole A.J., Wang Y., Cheng Y.Y., Hesselson D., Roelofs S.H., Neely G.G., Jang J.-H. (2023). Advanced pathophysiology mimicking lung models for accelerated drug discovery. Biomater. Res..

[63] Park S.B., Kim E.-A., Kim K.Y., Koh B. (2023). Induction of toxicity in human colon cells and organoids by size-and composition-dependent road dust. RSC Adv..

[64] Gałuszka-Bulaga A., Tkacz K., Węglarczyk K., Siedlar M., Baran J. (2023). Air pollution induces pyroptosis of human monocytes through activation of inflammasomes and Caspase-3-dependent pathways. J. Inflamm..

[65] Wang Y., Tang M. (2019). Integrative analysis of mRNAs, miRNAs and lncRNAs in urban particulate matter SRM 1648a-treated EA. hy926 human endothelial cells. Chemosphere.

[66] Pyambri M., Lacorte S., Jaumot J., Bedia C. (2023). Effects of Indoor Dust Exposure on Lung Cells: Association of Chemical Composition with Phenotypic and Lipid Changes in a 3D Lung Cancer Cell Model. Environ. Sci. Technol..

[67] Marín D., Orozco L.Y., Narváez D.M., Ortiz- Trujillo I.C., Molina F.J., Ramos C.D., Rodriguez-Villamizar L., Bangdiwala S.I., Morales O., Cuellar M. (2023). Characterization of the external exposome and its contribution to the clinical respiratory and early biological effects in children: The PROMESA cohort study protocol. PLoS ONE.

[68] Chen X., Luan M., Liu J., Yao Y., Li X., Wang T., Zhang H., Han Y., Lu X., Chen W. (2022). Risk factors in air pollution exposome contributing to higher levels of TNFα in COPD patients. Environ. Int..

[69] Patterson J.C., Joughin B.A., van de Kooij B., Lim D.C., Lauffenburger D.A., Yaffe M.B. (2019). ROS and oxidative stress are elevated in mitosis during asynchronous cell cycle progression and are exacerbated by mitotic arrest. Cell Syst..

[70] Seiler A., Schneider M., Förster H., Roth S., Wirth E.K., Culmsee C., Plesnila N., Kremmer E., Rådmark O., Wurst W. (2008). Glutathione peroxidase 4 senses and translates oxidative stress into 12/15-lipoxygenase dependent-and AIF-mediated cell death. Cell Metab..

[71] Wang B., Wang Y., Zhang J., Hu C., Jiang J., Li Y., Peng Z. (2023). ROS-induced lipid peroxidation modulates cell death outcome: Mechanisms behind apoptosis, autophagy, and ferroptosis. Arch. Toxicol..

[72] Lelieveld S., Wilson J., Dovrou E., Mishra A., Lakey P.S.J., Shiraiwa M., Pöschl U., Berkemeier T. (2021). Hydroxyl radical production by air pollutants in epithelial lining fluid governed by interconversion and scavenging of reactive oxygen species. Environ. Sci. Technol..

[73] He L., He T., Farrar S., Ji L., Liu T., Ma X. (2017). Antioxidants maintain cellular redox homeostasis by elimination of reactive oxygen species. Cell. Physiol. Biochem..

[74] Zorov D.B., Juhaszova M., Sollott S.J. (2014). Mitochondrial reactive oxygen species (ROS) and ROS-induced ROS release. Physiol. Rev..

[75] Tirichen H., Yaigoub H., Xu W., Wu C., Li R., Li Y. (2021). Mitochondrial reactive oxygen species and their contribution in chronic kidney disease progression through oxidative stress. Front. Physiol..

[76] Kowalczyk P., Sulejczak D., Kleczkowska P., Bukowska-Ośko I., Kucia M., Popiel M., Wietrak E., Kramkowski K., Wrzosek K., Kaczyńska K. (2021). Mitochondrial oxidative stress—A causative factor and therapeutic target in many diseases. Int. J. Mol. Sci..

[77] Peoples J.N., Saraf A., Ghazal N., Pham T.T., Kwong J.Q. (2019). Mitochondrial dysfunction and oxidative stress in heart disease. Exp. Mol. Med..

[78] Creed S., McKenzie M. (2019). Measurement of mitochondrial membrane potential with the fluorescent dye tetramethylrhodamine methyl ester (TMRM). Cancer Metab. Methods Protoc..

[79] Tang D., Chen X., Kang R., Kroemer G. (2021). Ferroptosis: Molecular mechanisms and health implications. Cell Res..

[80] Jiang X., Stockwell B.R., Conrad M. (2021). Ferroptosis: Mechanisms, biology and role in disease. Nat. Rev. Mol. Cell Biol..

[81] Liang D., Minikes A.M., Jiang X. (2022). Ferroptosis at the intersection of lipid metabolism and cellular signaling. Mol. Cell.

[82] Morris G., Gevezova M., Sarafian V., Maes M. (2022). Redox regulation of the immune response. Cell. Mol. Immunol..

[83] Su L.-J., Zhang J.-H., Gomez H., Murugan R., Hong X., Xu D., Jiang F., Peng Z.-Y. (2019). Reactive oxygen species-induced lipid peroxidation in apoptosis, autophagy, and ferroptosis. Oxid. Med. Cell. Longev..

[84] Wu Y., Wang J., Zhao T., Sun M., Xu M., Che S., Pan Z., Wu C., Shen L. (2024). Polystyrenenanoplastics lead to ferroptosis in the lungs. J. Adv. Res..

[85] Sinenko S.A., Starkova T.Y., Kuzmin A.A., Tomilin A.N. (2021). Physiological signaling functions of reactive oxygen species in stem cells: From flies to man. Front. Cell Dev. Biol..

[86] Morgan M.J., Liu Z. (2011). Crosstalk of reactive oxygen species and NF-κB signaling. Cell Res..

[87] Juan C.A., Pérez de la Lastra J.M., Plou F.J., Pérez-Lebeña E. (2021). The chemistry of reactive oxygen species (ROS) revisited: Outlining their role in biological macromolecules (DNA, lipids and proteins) and induced pathologies. Int. J. Mol. Sci..

[88] Bajpai V.K., Alam M.B., Quan K.T., Kwon K.-R., Ju M.-K., Choi H.-J., Lee J.S., Yoon J.-I., Majumder R., Rather I.A. (2017). Antioxidant efficacy and the upregulation of Nrf2-mediated HO-1 expression by (+)-lariciresinol, a lignan isolated from Rubia philippinensis, through the activation of p38. Sci. Rep..

[89] Redza-Dutordoir M., Averill-Bates D.A. (2016). Activation of apoptosis signalling pathways by reactive oxygen species. Biochim. Biophys. Acta Mol. Cell Res..

[90] Eruslanov E., Kusmartsev S. (2009). Identification of ROS Using Oxidized DCFDA and Flow-Cytometry. Methods Mol. Biol..

[91] Murphy M.P., Bayir H., Belousov V., Chang C.J., Davies K.J.A., Davies M.J., Dick T.P., Finkel T., Forman H.J., Janssen-Heininger Y. (2022). Guidelines for measuring reactive oxygen species and oxidative damage in cells and in vivo. Nat. Metab..

[92] Kim H., Xue X. (2020). Detection of Total Reactive Oxygen Species in Adherent Cells by 2′,7′-Dichlorodihydrofluorescein Diacetate Staining. J. Vis. Exp..

[93] Ameziane-El-Hassani R., Dupuy C. (2013). Detection of Intracellular Reactive Oxygen Species (CM-H2DCFDA). Bio-Protocol.

[94] Schieber M., Chandel N.S. (2014). ROS function in redox signaling and oxidative stress. Curr. Biol..

[95] Lennicke C., Cochemé H.M. (2021). Redox metabolism: ROS as specific molecular regulators of cell signaling and function. Mol. Cell.

[96] Kovac S., Angelova P.R., Holmström K.M., Zhang Y., Dinkova-Kostova A.T., Abramov A.Y. (2015). Nrf2 regulates ROS production by mitochondria and NADPH oxidase. Biochim. Biophys. Acta (BBA) Gen. Subj..

[97] Kasai S., Shimizu S., Tatara Y., Mimura J., Itoh K. (2020). Regulation of Nrf2 by mitochondrial reactive oxygen species in physiology and pathology. Biomolecules.

[98] Brown D.M., Donaldson K., Stone V. (2010). Nuclear translocation of Nrf2 and expression of antioxidant defence genes in THP-1 cells exposed to carbon nanotubes. J. Biomed. Nanotechnol..

[99] Hsieh C.-Y., Hsiao H.-Y., Wu W.-Y., Liu C.-A., Tsai Y.-C., Chao Y.-J., Wang D.L., Hsieh H.-J. (2009). Regulation of shear-induced nuclear translocation of the Nrf2 transcription factor in endothelial cells. J. Biomed. Sci..

[100] Loboda A., Damulewicz M., Pyza E., Jozkowicz A., Dulak J. (2016). Role of Nrf2/HO-1 system in development, oxidative stress response and diseases: An evolutionarily conserved mechanism. Cell. Mol. Life Sci..

[101] Zhang Q., Liu J., Duan H., Li R., Peng W., Wu C. (2021). Activation of Nrf2/HO-1 signaling: An important molecular mechanism of herbal medicine in the treatment of atherosclerosis via the protection of vascular endothelial cells from oxidative stress. J. Adv. Res..

[102] Kim J.-S., Oh J.-M., Choi H., Kim S.W., Kim S.W., Kim B.G., Cho J.H., Lee J., Lee D.C. (2020). Activation of the Nrf2/HO-1 pathway by curcumin inhibits oxidative stress in human nasal fibroblasts exposed to urban particulate matter. BMC Complement. Med. Ther..

[103] Pardo M., Qiu X., Zimmermann R., Rudich Y. (2020). Particulate Matter Toxicity Is Nrf2 and Mitochondria Dependent: The Roles of Metals and Polycyclic Aromatic Hydrocarbons. Chem. Res. Toxicol..

[104] Gui J., Wang L., Liu J., Luo H., Huang D., Yang X., Song H., Han Z., Meng L., Ding R. (2024). Ambient particulate matter exposure induces ferroptosis in hippocampal cells through the GSK3B/Nrf2/GPX4 pathway. Free Radic. Biol. Med..

[105] Chiang S.-K., Chen S.-E., Chang L.-C. (2021). The Role of HO-1 and Its Crosstalk with Oxidative Stress in Cancer Cell Survival. Cells.

[106] Piras S., Furfaro A.L., Brondolo L., Passalacqua M., Marinari U.M., Pronzato M.A., Nitti M. (2017). Differentiation impairs Bach1 dependent HO-1 activation and increases sensitivity to oxidative stress in SH-SY5Y neuroblastoma cells. Sci. Rep..

[107] Doll S., Proneth B., Tyurina Y.Y., Panzilius E., Kobayashi S., Ingold I., Irmler M., Beckers J., Aichler M., Walch A. (2017). ACSL4 dictates ferroptosis sensitivity by shaping cellular lipid composition. Nat. Chem. Biol..

[108] Lee H., Gan B. (2022). Ferroptosis execution: Is it all about ACSL4?. Cell Chem. Biol..

[109] Koppula P., Zhuang L., Gan B. (2021). Cystine transporter SLC7A11/xCT in cancer: Ferroptosis, nutrient dependency, and cancer therapy. Protein Cell.

[110] Yan Y., Teng H., Hang Q., Kondiparthi L., Lei G., Horbath A., Liu X., Mao C., Wu S., Zhuang L. (2023). SLC7A11 expression level dictates differential responses to oxidative stress in cancer cells. Nat. Commun..

[111] Fournier M., Monin A., Ferrari C., Baumann P.S., Conus P., Do K. (2017). Implication of the glutamate-cystine antiporter xCT in schizophrenia cases linked to impaired GSH synthesis. NPJ Schizophr..

[112] Liu J., Xia X., Huang P. (2020). xCT: A Critical Molecule That Links Cancer Metabolism to Redox Signaling. Mol. Ther..

[113] Jyotsana N., Ta K.T., DelGiorno K.E. (2022). The Role of Cystine/Glutamate Antiporter SLC7A11/xCT in the Pathophysiology of Cancer. Front. Oncol..

[114] Lim J.K.M., Delaidelli A., Minaker S.W., Zhang H.-F., Colovic M., Yang H., Negri G.L., von Karstedt S., Lockwood W.W., Schaffer P. (2019). Cystine/glutamate antiporter xCT (SLC7A11) facilitates oncogenic RAS transformation by preserving intracellular redox balance. Proc. Natl. Acad. Sci. USA.

[115] Shin C.-S., Mishra P., Watrous J.D., Carelli V., D’Aurelio M., Jain M., Chan D.C. (2017). The glutamate/cystine xCT antiporter antagonizes glutamine metabolism and reduces nutrient flexibility. Nat. Commun..

[116] Ma T., Du J., Zhang Y., Wang Y., Wang B., Zhang T. (2022). GPX4-independent ferroptosis—A new strategy in disease’s therapy. Cell Death Discov..

[117] Xu C., Sun S., Johnson T., Qi R., Zhang S., Zhang J., Yang K. (2021). The glutathione peroxidase Gpx4 prevents lipid peroxidation and ferroptosis to sustain Treg cell activation and suppression of antitumor immunity. Cell Rep..

[118] Chen X., Yu C., Kang R., Tang D. (2020). Iron Metabolism in Ferroptosis. Front. Cell Dev. Biol..

[119] Rochette L., Dogon G., Rigal E., Zeller M., Cottin Y., Vergely C. (2022). Lipid Peroxidation and Iron Metabolism: Two Corner Stones in the Homeostasis Control of Ferroptosis. Int. J. Mol. Sci..

[120] Hao H., Wang Y., Zhu Q., Zhang H., Rosenberg A., Schwartz J., Amini H., van Donkelaar A., Martin R., Liu P. (2023). National Cohort Study of Long-Term Exposure to PM2. 5 Components and Mortality in Medicare American Older Adults. Environ. Sci. Technol..

[121] Yang Z., Mahendran R., Yu P., Xu R., Yu W., Godellawattage S., Li S., Guo Y. (2022). Health effects of long-term exposure to ambient PM2. 5 in Asia-Pacific: A systematic review of cohort studies. Curr. Environ. Health Rep..

[122] Aghaei-Zarch S.M., Nia A.H.S., Nouri M., Mousavinasab F., Najafi S., Bagheri-Mohammadi S., Aghaei-Zarch F., Toolabi A., Rasoulzadeh H., Ghanavi J. (2023). The impact of particulate matters on apoptosis in various organs: Mechanistic and therapeutic perspectives. Biomed. Pharmacother..

[123] Lu Y., Cao M., Li F., Tian M., Ren H., Chi Q., Huang Q. (2023). Atmospheric PM2. 5 induce autophagy and autophagic flux blockage in HUVEC cells via ROS/TXNIP signaling: Important role of metal components. J. Hazard. Mater..

[124] Shan X., Liu L., Li G., Xu K., Liu B., Jiang W. (2021). PM2. 5 and the typical components cause organelle damage, apoptosis and necrosis: Role of reactive oxygen species. Sci. Total Environ..

[125] Sargent M., Harte R., Harrington C. (2002). Guidelines for Achieving High Accuracy in Isotope Dilution Mass Spectrometry (IDMS).

[126] Greenberg R.R., Bode P., De Nadai Fernandes E.A. (2011). Neutron activation analysis: A primary method of measurement. Spectrochim. Acta Part B At. Spectrosc..

[127] Baek S.-Y., Lim D.K., Han J., Lee S., Kim B. (2021). Method development for accurate determination of eight polycyclic aromatic hydrocarbons in extruded high-impact polystyrene. Chemosphere.

[128] Shiraiwa T., Fujino N. (1966). Theoretical Calculation of Fluorescent X-ray Intensities in Fluorescent X-ray Spectrochemical Analysis. Jpn. J. Appl. Phys..

