# Supplementary material for: Particulate Matter Induces Oxidative Stress and Ferroptosis in Human Lung Epithelial Cells
Source: Toxics. 2024 Feb 19;12(2):161. doi: 10.3390/toxics12020161 (PMC10893167; doi:10.3390/toxics12020161)
Supplement: Supplementary file 1 [file toxics-12-00161-s001.zip › toxics-2847913-supplementary.pdf]

toxics

Supplementary Materials: Particulate Matter Induces Oxidative Stress and Ferroptosis in Human Lung Epithelial Cells

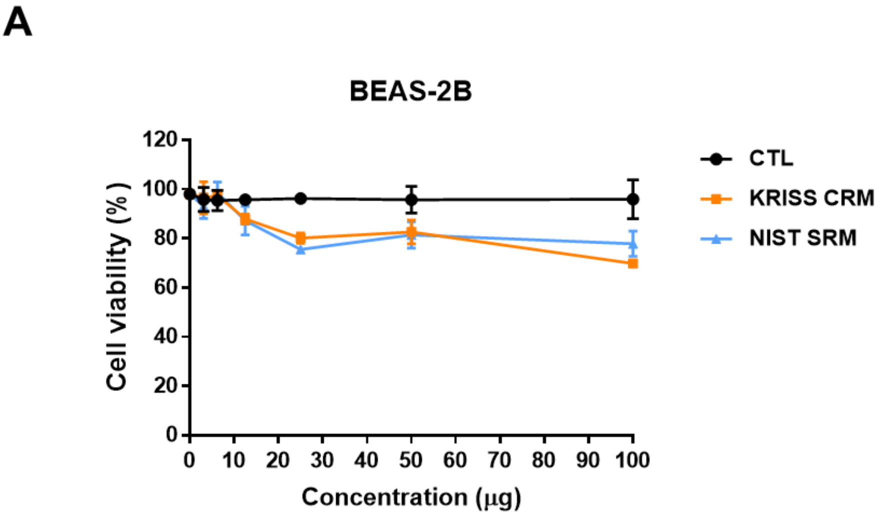

Figure S1. The cell viability of KRISS CRM 109-02-004 and NIST SRM 1648a. (A) After exposure to KRISS CRM and NIST SRM, cells were treated with concentrations of 0, 50, 100, and 200 μg/ml for 48 hours, followed by measurement of cell proliferation using MTS assay.

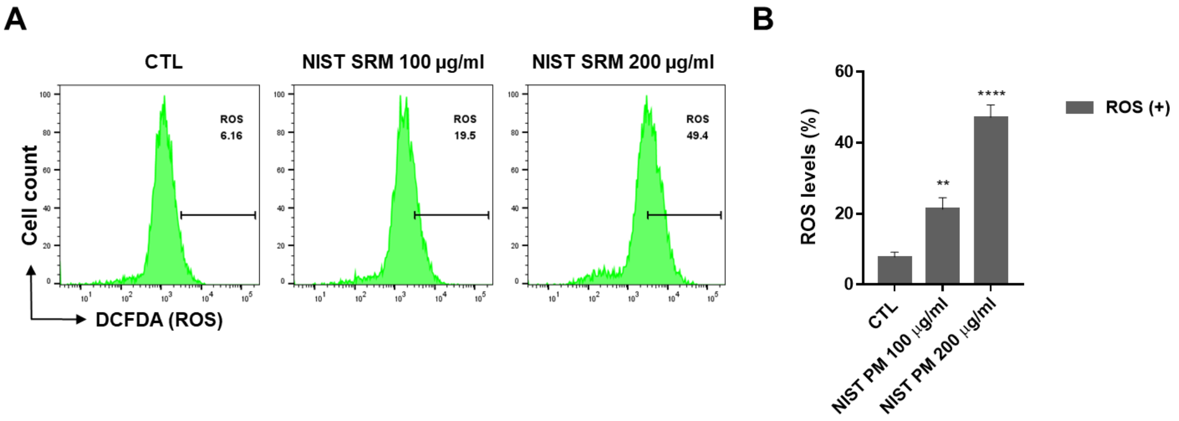

Figure S2. Cellular ROS were induced by NIST SRM in BEAS-2B cells. (A) ROS production was analyzed via H2DCFDA staining-based (10 μM) flow cytometry. ROS levels were assessed by DCFH-DA after 48 hours of exposure to 100 μg and 200 μg of NIST SRM in BEAS-2B cells. (B) Quantification of ROS levels. Statistical significance is indicated as: ** $p < 0.01$, **** $p \leq 0.0001$.

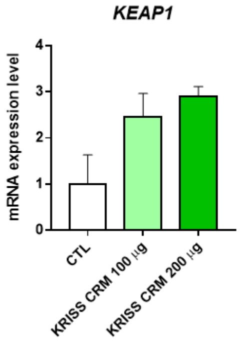

Figure S3. The mRNA expression level of Keap1. Quantification of mRNA levels of Keap1 was performed by RT-qPCR analysis.

Information on the urban particulate matter CRM, KRISS CRM 109-02-004.

Table S1 provides the certified values of the CRM. These values were obtained through established measurement techniques at KRISS. Sb, Ca, Cu, Pb, Mg, and Sn mass fractions were determined using isotope dilution inductively coupled plasma mass spectrometry (ID-ICP/MS) [1], while Cr and Co were determined using standard comparator (SC) instrumental neutron activation analysis (INAA) [2]. Analysis involved examining at least ten systematically selected bottles from a sample batch to establish certified values. Mass fractions of polycyclic aromatic hydrocarbons (PAHs) were determined via isotope dilution gas chromatography-mass spectrometry (GC-MS) [3] using accelerated solvent extraction (ASE) with dichloromethane. Uncertainties in the certified values account for between-bottle inhomogeneity. Sample sizes for analysis varied, with at least 10 mg or 50 mg for elements analyzed by ID-ICP/MS and SC-INAA, respectively, and 150 mg for PAHs. The certified values are reported on a dry-mass basis. Moisture content corrections were performed by drying at least three moisture measurement samples, each about 100 mg, in a desiccator containing P2O5 as a desiccant for seven days (168 hours).

Table S1. Certified values of KRISS CRM 109-02-004, urban particulate matter.

Analyte	Certified value[1]	Analytical method
Antimony (Sb)	0.0679 ± 0.0030 g/kg	IDICP/MS
Calcium (Ca)	39.5 ± 2.4 g/kg	ID ICP/MS
Chromium (Cr)	0.307 ± 0.014 g/kg	SC INAA
Cobalt (Co)	0.01996 ± 0.00070 g/kg	SC INAA
Copper (Cu)	4.01 ± 0.30 g/kg	ID ICP/MS
Lead (Pb)	0.293 ± 0.013 g/kg	ID ICP/MS
Magnesium (Mg)	9.43 ± 0.54 g/kg	ID ICP/MS
Tin (Sn)	0.191 ± 0.012 g/kg	ID ICP/MS
Zinc (Zn)	8.17 ± 0.44 g/kg	ID ICP/MS
Benz(a)anthracene	0.494 ± 0.044 mg/kg	ID GC-MS
Benzo(a)pyrene	0.354 ± 0.032 mg/kg	ID GC-MS
Benzo(b)fluoranthene	1.24 ± 0.12 mg/kg	ID GC-MS
Benzo(e)pyrene	0.89 ± 0.10 mg/kg	ID GC-MS
Benzo(g,h,i)perylene	1.11 ± 0.12 mg/kg	ID GC-MS
Benzo(j)fluoranthene	0.466 ± 0.046 mg/kg	ID GC-MS
Benzo(k)fluoranthene	0.420 ± 0.045 mg/kg	ID GC-MS
Chrysene	1.019 ± 0.097 mg/kg	ID GC-MS

Indeno(1,2,3-cd)pyrene	0.742 ± 0.087 mg/kg	ID GC-MS
Triphenylene	0.399 ± 0.039 mg/kg	ID GC-MS

1. Numbers after ± are the expanded uncertainties stated at the 95 % level of confidence.

The approximate mass fractions of the remaining major elements were determined using either ICP/MS or X-ray fluorescence (XRF) spectrometry. Specifically, an Agilent 8800 triple quadrupole ICP/MS operated in MS/MS mode with H_2 as a reaction gas was utilized. External calibration curves were employed for semi-quantitative analysis of these major elements in the urban particulate matter CRM. For semi-quantitative XRF analysis, a ZSX Primus II WD-XRF spectrometer (Rigaku, Japan) was employed. This instrument utilized eight different analyzing crystals (LiF200, PET, Ge, RX-25, RX-40, RX-45, RX-61F, and RX-75) along with two detectors, scintillation, and gas flow counters, to analyze various elements. Samples were formed into discs for analysis, and the fundamental parameter method was utilized [4].

Table S2. Approximate contents of elements in KRISS CRM 109-02-004, urban particulate matter.

Analyte	ICP/MS (g/kg)	WD-XRF (g/kg)
Sodium (Na)	10.7	13.1
Aluminium (Al)	37.0	45.5
Silicon (Si)	-	140
Potassium (K)	17.7	15.1
Titanium (Ti)	-	4.1
Manganese (Mn)	1.5	1.2
Iron (Fe)	99.0	81.2

Information on the primer sequences and corresponding genes.

Table S3. The primer sequence of genes used in RT-qPCR

Gene name	Sequences
xCT/SLC7A11	F: 5'-TCCTGCTTTGGCTCCATGAACG-3'<br>R: 5'-AGAGGAGTGTGCTTGCGGACAT-3'
FTL	F: 5'-TACGAGCGTCTCCTGAAGATGC-3'<br>R: 5'-GGTTCAGCTTTTTCTCCAGGGC-3'
NQO1	F: 5'-TGGCTCCATGTACTCTCTGC-3'<br>R: 5'-CAGAAATGCAGAATGCCACT-3'
TRIM16	F: 5'-GGCTGAAGGATAAACTCTCGGG-3'<br>R: 5'-CGCTGAACAACGGCAGACACTT-3'
CYP1B1	F: 5'-GCCACTATCACTGACATCTTCGG-3'<br>R: 5'-CACGACCTGATCCAATTCTGCC-3'
FTH1	F: 5'-TGAAGCTGCAGAACCAACGAGG-3'<br>R: 5'-GCACACTCCATTGCATTCAGCC-3'

GPX4	F: 5'-ACAAGAACGGCTGCGTGGTGAA-3'<br>R: 5'-GCCACACACTTGTGGAGCTAGA-3'
SOD2	F: 5'-CTGGACAAACCTCAGCCCTAAC-3'<br>R: 5'-AACCTGAGCCTTGGACACCAAC-3'
NOX4	F: 5'-GCCAGAGTATCACTACCTCCAC-3'<br>R: 5'-CTCGGAGGTAAGCCAAGAGTGT-3'
ACSL4	F: 5'-GCTATCTCCTCAGACACACCGA-3'<br>R: 5'-AGGTGCTCCAACTCTGCCAGTA-3'
CHAC1	F: 5'-GTGGTGACGCTCCTTGAAGATC-3'<br>R: 5'-GAAGGTGACCTCCTTGGTATCG-3'
RGS4	F: 5'-ACATCGGCTAGGTTTCCTGCTG-3'<br>R: 5'-CAGGTTTTCCAGTGATTCAGCCC-3'
CAV1	F: 5'-CCAAGGAGATCGACCTGGTCAA-3'<br>R: 5'-GCCGTCAAAACTGTGTGTCCCT-3'
NRF2	F: 5'-CACATCCAGTCAGAAACCAGTGG-3'<br>R: 5'-GGAATGTCTGCGCCAAAAGCTG-3'
CAT	F: 5'-ACGAGATGGCACACTTTGACAG-3'<br>R: 5'-TGGGTTTCTCTTCTGGCTATGG-3'

1. Guidelines for Achieving High Accuracy in Isotope Dilution Mass Spectrometry (IDMS). *Guidel. Achiev. High Accuracy Isot. Dilution Mass Spectrom.* 2002, 1–34.

2. Greenberg, R.R.; Bode, P.; De Nadai Fernandes, E.A. Neutron activation analysis: A primary method of measurement. *Spectrochim. Acta Part B At. Spectrosc.* **2011**, *66*, 193–241, doi:10.1016/j.sab.2010.12.011.

3. Baek, S.-Y.; Lim, D.K.; Han, J.; Lee, S.; Kim, B. Method development for accurate determination of eight polycyclic aromatic hydrocarbons in extruded high-impact polystyrene. *Chemosphere* **2021**, *272*, 129909, doi:10.1016/j.chemosphere.2021.129909.

4. Shiraiwa, T.; Fujino, N. Theoretical Calculation of Fluorescent X-Ray Intensities in Fluorescent X-Ray Spectrochemical Analysis. *Jpn. J. Appl. Phys.* **1966**, *5*, 886, doi:10.1143/jjap.5.886.
